# A Validated LC-MS/MS Method for Simultaneous Determination of Six* Aconitum* Alkaloids and Seven Ginsenosides in Rat Plasma and Application to Pharmacokinetics of Shen-Fu Prescription

**DOI:** 10.1155/2018/5107083

**Published:** 2018-06-28

**Authors:** Huizi Ouyang, Fang Liu, Zhidan Tang, Xiaopeng Chen, Fang Bo, Huaming Liu, Yanxu Chang, Jun He

**Affiliations:** ^1^First Teaching Hospital of Tianjin University of Traditional Chinese Medicine, Tianjin 300193, China; ^2^Tianjin State Key Laboratory of Modern Chinese Medicine, Tianjin University of Traditional Chinese Medicine, Tianjin 300193, China

## Abstract

A sensitive and reliable LC-MS/MS method has been developed and validated for simultaneous determination of six* Aconitum* alkaloids (aconitine, hypaconitine, mesaconitine, benzoylaconitine, benzoylhypacoitine, and benzoylmesaconine) and seven ginsenosides (Rb_1_, Rb_2_, Rc, Rd, Re, Rf, and Rg_1_) in rat plasma after oral administration of Shen-Fu prescription. Psoralen was selected as internal standard (IS). Protein precipitation with methanol was used in sample preparation. The chromatographic separation was achieved on a CORTECS™ C18 column with 0.1% formic acid aqueous solution and acetonitrile as mobile phase. The flow rate was 0.3 mL/min. The detection was performed on a tandem mass system with an electrospray ionization (ESI) source in the positive ionization and multiple-reaction monitoring (MRM) mode. The calibration curves of six* Aconitum* alkaloids and seven ginsenosides were linear over the range of 0.1-50 and 1-500 ng/mL, respectively. The extraction recoveries of the analytes in plasma samples ranged from 64.2 to 94.1%. Meanwhile, the intra- and interday precision of the analytes were less than 14.3%, and the accuracy was in the range of −14.2% to 9.8%. The developed method was successfully applied to the pharmacokinetics of six* Aconitum* alkaloids and seven ginsenosides in rat plasma after oral administration of Shen-Fu prescription.

## 1. Introduction

Shen-Fu prescription, which was derived from traditional Chinese medicine, was first documented in a Chinese medical book Jisheng Fang more than 7 centuries ago. Shen-Fu prescription is composed of Radix* Aconite Lateralis* Preparata (*Fuzi* in Chinese) and Radix* Ginseng Rubra* (*Hongshen* in Chinese). It was simpler and more effective than other traditional Chinese herbal prescriptions in the treatment of heart failure and shock. Therefore, it is not only commonly used as herbal medicines in Asian countries, but also successfully developed into a serial of modern pharmaceutical dosage forms, such as injection or oral formulation. Pharmacological research demonstrates that the mechanism of its bioactivity including blocking sodium channels and delaying apoptosis of myocardiocytes [1–3].

**Figure 1:**
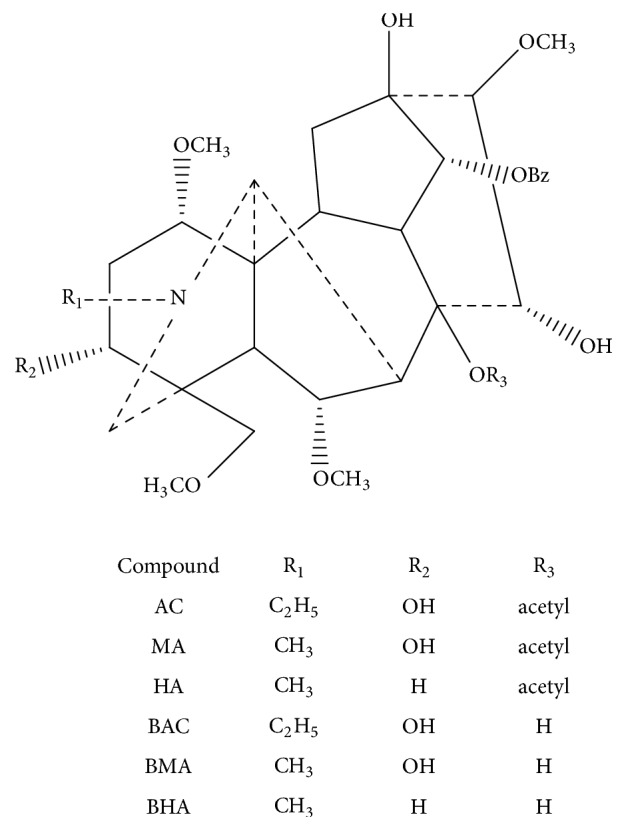
The chemical structures of AC, MA, HA, BAC, BMA, and BHA.

*Fuz*i, the lateral roots of* Aconitum carmichaelii* Debx., exhibits pharmacological effects including strengthening cardiotonic function and resisting inflammation [4]. Moreover, water-soluble components of* Fuzi* show prospects for the control of cancer in a synergistic manner [5]. The main bioactive components of* Fuzi* are monoester-diterpenoid alkaloids (MDAs) such as benzoylaconine (BAC), benzoylmesaconine (BMA), and benzoylhypaconine (BHA) and diester-diterpenoid alkaloids (DDAs) such as aconitine (AC), mesaconitine (MA), and hypaconitine (HA) [6–8]. The structures of alkaloids are shown in**Figure 1**. However, the diester-diterpenoid* Aconitum* alkaloids have a narrow therapeutic window, which means suitable dose is beneficial to medical treatment while overdose could be hazardous [9–11].

**Figure 2:**
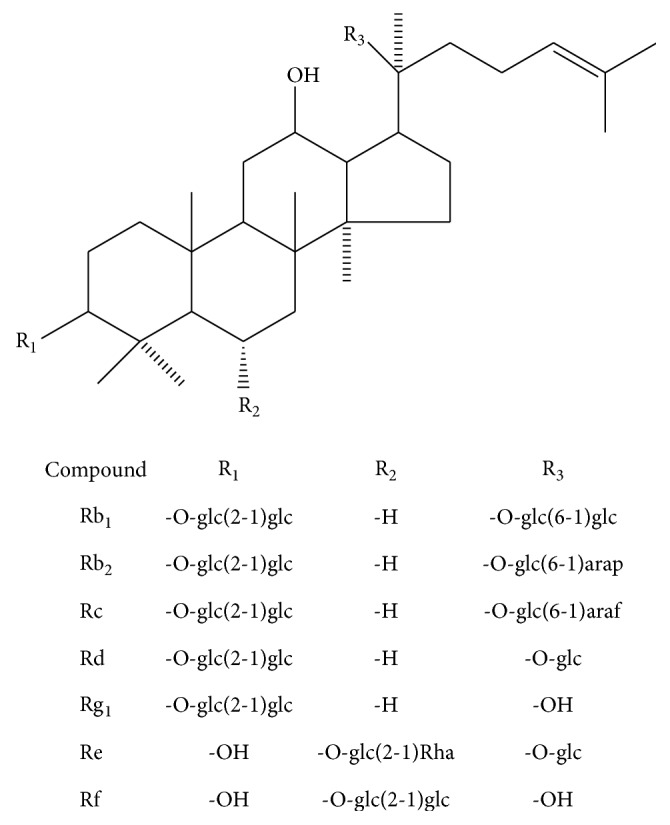
The chemical structures of Rb_1_, Rb_2_, Rc, Rd, Re, Rf, and Rg_1_.

The bioactive components of Hongshen are a large number of triterpenoid saponins known as ginsenosides. According to the structures, the ginsenosides could be classified into two major groups, that is, 20(S)-protopanaxadiol type (ppd-type) and 20(S)-protopanaxatriol type (ppt-type). The major ginsenosides present in Hongshen include the ppd-type Rb_1_, Rb_2_, Rc, and Rd and the ppt-type Re, Rf, and Rg_1_ [12]. The structures are shown in**Figure 2**. The publications report the bioactivity of ginsenosides including cardiovascular protection, oxidized free radicals scavenging, and cell immunity regulation [13–15]. Furthermore, the ginsenosides could change the absorption of* Aconitum* alkaloids to reduce the toxicity [16]. Some pharmacological studies have indicated that these ginsenosides inhibit the cardiac toxicity of* Aconitum* alkaloids by regulating the mitochondrial energy metabolism and promoting the antioxidant activities [17–19].

Based on the publications [20–25], the curative effect of Shen-Fu prescription is an integrative effect of ginsenosides and alkaloids. Thus, a simple and sensitive analytical method to determine AC, HA, MA, BAC, BHC, BMA, Rb_1_, Rb_2_, Rc, Rd, Re, Rf, and Rg_1_ in plasma is important for the pharmacokinetic behavior of Shen-Fu prescription. Some manuscripts describe the determination of Shen-Fu composition in plasma using HPLC and LC-MS, but their application to pharmacokinetics study was mainly focused on a class of ingredients, such as alkaloids or ginsenosides [26–28]. Because of low sensitivity and poor selectivity, only one manuscript reported the analysis of both ginsenosides (Rg_1_, Rb_1_, and Rc) and Aconitum alkaloids (BMA and fuziline) in biological samples after intravenous administration of Shen-Fu injection [29]. Despite this, it is still challenging to achieve simultaneous determination of MDAs, DDAs, ppd-type, and ppt-type ginsenosides in biological samples because of the multiple components with different physicochemical properties and polarities. In this paper, an LC-MS/MS method for the simultaneous determination of six* Aconitum* alkaloids and seven ginsenosides in rat plasma was established and validated. The method was applied to the pharmacokinetic study after oral administration of Shen-Fu prescription. It is very necessary to gain an insight into the pharmacokinetic characters of these alkaloids and ginsenosides, which provides valuable information to the therapy, further preclinical and clinical studies.

## 2. Materials and Methods

### 2.1. Chemicals, Reagents, and Apparatus

The standard compounds of AC, HA, MA, BAC, BHC, BMA, Rb_1_, Rb_2_, Rc, Rd, Re, Rf, Rg_1_, and psoralen (purity ≥98%, confirmed by LC-MS/MS, respectively) were purchased from Chengdu Must Bio-Technology Co., Ltd. (Chengdu, China).* Fuzi* and* Hongshen* were purchased from Hebei Anguo Medicina Material Company (Hebei, China) and authenticated by Professor Tian-xiang Li (Tianjin University of Traditional Chinese Medicine, Tianjin, China). Acetonitrile and methanol of HPLC grade were obtained from Merck (Darmstadt, Germany). Formic acid of HPLC grade was purchased from ROE SCIENTIFIC INC (Newark, USA). Ultrapure water was purified by a Milli-Q water purification system (Millipore, Milford, MA, USA).

The LC-MS/MS system consisted of an Agilent 1200 HPLC system coupled to an Agilent 6430 triple-quadrupole mass spectrometer equipped with ESI source. All the operations and analysis of data were performed by Mass Hunter Workstation Software from Agilent Technologies (version B.04.00).

### 2.2. Chromatographic and Mass Spectrometry Condition

The chromatographic separation was achieved on a CORTECS™ C18 column (2.1×50mm, 2.7 *μ*m). The mobile phase consisted of water with 0.1% (v/v) formic acid (A) and acetonitrile (B) using a gradient elution of 13-26% B at 0-7 min, 26% B at 7-15 min. The flow rate was 0.3 mL/min. The column temperature was set at 30°C. The sample injection volume was 5 *μ*L.

All analytes were confirmed and quantified by tandem mass spectrometry operating in the electrospray positive ionization mode (ESI^+^) with multiple-reaction monitoring (MRM) mode. The MS parameters were optimized and set as follows: capillary voltage at 500 V, nebulizer at 20 psi, drying gas flow rate at 9 L/min, and temperature at 300°C. Quantitative parameters are listed in**Table 1**.

### 2.3. Preparation of Shen-Fu Prescription

*Fuzi* and* Hongshen* (weight/weight=1:1) were extracted with 70% ethanol, respectively. After evaporated to dryness, two extracts were mixed well for preparing Shen-Fu prescription. The contents of the six alkaloids and seven ginsenosides in Shen-Fu prescription were determined by LC-MS/MS. It contained AC 0.02 mg/g, HA 0.28 mg/g, MA 0.03 mg/g, BAC 0.12 mg/g, BHA 0.28 mg/g, BMA 0.44 mg/g, Rb_1_ 0.96 mg/g, Rb_2_ 1.43 mg/g, Rc 0.66 mg/g, Re 0.17 mg/g, Rd 0.19 mg/g, Rf 0.24 mg/g, and Rg_1_ 0.14 mg/g. All extracts were kept at 4°C for further oral administration to rats.

### 2.4. Preparation of Calibration Standards and Quality Control Samples

The stock solution of AC, HA, MA, BAC, BHA, BMA, Rb_1_, Rb_2_, Rc, Rd, Re, Rf, and Rg_1_ was prepared in methanol at a concentration of 100 *μ*g/mL, respectively. Appropriate aliquots of the stock solutions were calculated and mixed together to prepare a mixed stock solution and then diluted with methanol to achieve working solutions of the desired concentrations. Psoralen was prepared in methanol as IS solution at 1 *μ*g/mL. All solutions were preserved at 4°C and were brought to room temperature before use. The calibration standard solutions were prepared by adding appropriate amounts of the working solutions to 100 *μ*L blank rat plasma with IS to gain linear range of 0.1-50 ng/mL for AC, HA, MA, BAC, BHA, and BMA and 1-500 ng/mL for Rb_1_, Rb_2_, Rc, Rd, Re, Rf, and Rg_1_. The quality control (QC) samples were prepared at 0.2, 5, and 50 ng/mL for AC, HA, MA, BAC, BHA, and BMA and 2, 50, and 500 ng/mL for Rb_1_, Rb_2_, Rc, Rd, Re, Rf, and Rg_1_.

### 2.5. Plasma Sample Preparation

The plasma samples were allowed to thaw gradually to room temperature before processing. One hundred microliter plasma was mixed with 20 *μ*L IS working solution and 20 *μ*L methanol (equal volume of the corresponding working solution added to calibration curve and QC samples) by vortexing for 30 s in a centrifuge tube. The sample was added to 1 mL methanol and then shaked for 5 min. After centrifugation at 14,000 rpm for 10 min, the supernatant was collected into a centrifuge tube and dried gently under the constant flow of nitrogen. The obtained residue was redissolved in 100 *μ*L 50% methanol by vortexing and then centrifuged at 14,000 rpm under 4°C for 10 min. Ten-microliter supernatant was injected into the LC-MS/MS system for analysis.

### 2.6. Method Validation

The specificity was evaluated by comparing chromatograms of blank plasma samples from six different rats with QC samples and plasma samples after oral administration of Shen-Fu prescription.

For the linearity, the calibration curves were generated from seven concentrations by assaying standard plasma samples in duplicate on 3 different days. Series of samples were evaluated by least-square regression using 1/*x*^*2*^ as a weighting factor. The lower limit of quantification (LLOQ) could meet the analytical requirement of signal-to-noise ratio (S/N) above 10, accuracy within ±20%, and precision below 20%.

The precision and accuracy were assessed by assaying four batches of QC samples at LLOQ, low, medium, and high concentration levels (each including six replicates) on three separate days. The intra- and interday precisions, validated by relative standard deviation (RSD), were required to be less than 15%. The accuracy, estimated by comparing the measured concentration with its true value, was required within ±15%.

The recovery of the analytes and IS were calculated by comparing peak areas of extracted plasma samples with postextracted spiked samples at the same theoretical concentrations.

The matrix effect was assessed by comparing the peak area of the analytes in postextracted spiked samples with standard solutions at the same theoretical concentrations.

The stability of analytes in rat plasma was assessed by analyzing QC samples in triplicate at different conditions: at room temperature for 3 h, in autosampler after preparation for 4 h, at −20°C for 7 days, and three freeze-thaw cycles, respectively.

### 2.7. Application

Eight Sprague-Dawley rats (210-230 g) were purchased from Beijing Weitong Lihua Biotechnology Co. Ltd. (Beijing, China). The animals were kept in a stable environment by controlling temperature between 23 and 26°C and maintaining relative humidity of 40-60%. After 7 days of acclimation, the rats were fasted for 12 h but provided with water freely before the experiments. Each rat was given Shen-Fu prescription by oral administration at a dose of 4.75 g/kg. Blood samples (250 *μ*L) were collected from fossa orbitalis vein into heparinized polythene tubes at the designated time points (0, 0.03, 0.08, 0.17, 0.25, 0.5, 1, 2, 4, 6, 8, 10, 12, 24, 36, 48, 60, 72, and 96 h). After centrifugation at 6000 rpm for 10 min, the supernatant was transferred into the clean centrifuge tube and stored at −70°C until analysis. Pharmacokinetic parameters were calculated by software “Drug and Statistics 2.0” (DAS 2.0) (Medical College of Wannan, China).

## 3. Results and Discussion

### 3.1. Mass Spectrometry

The response of the analytes and IS in ESI source were evaluated by recording the full-scan mass spectrum. Positive ion mode was chosen as stable and strong MS signal observed.

### 3.2. Optimization of the Chromatographic Condition

**Figure 3:**
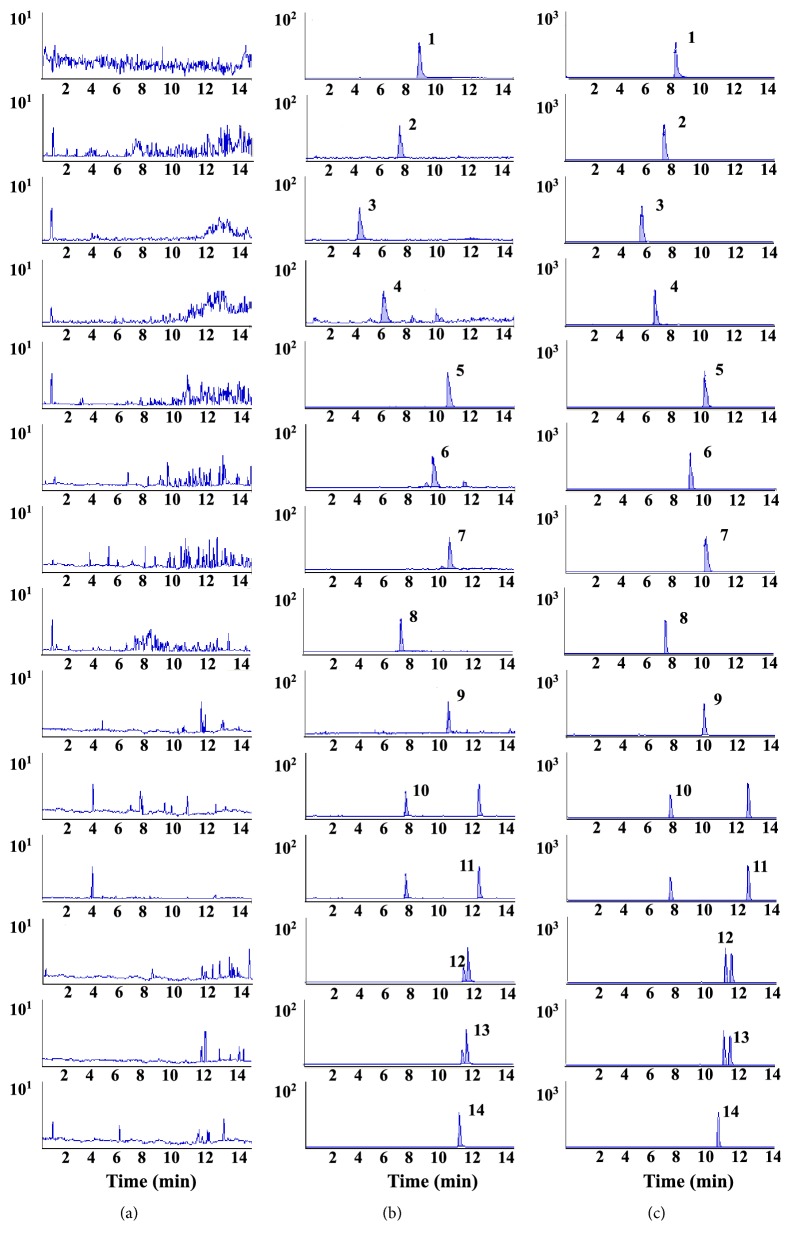
MRM chromatograms of IS (1), BHA (2), BMA (3), BAC (4), HA (5), MA (6), AC (7), Rg_1_ (8), Rf (9), Re (10), Rd (11), Rc(12), Rb_2_ (13), and Rb_1_ (14). (a) Blank plasma; (b) blank plasma spiked with the 13 compounds and IS; (c) a rat plasma sample after administration of Shen-Fu prescription.

The chromatographic conditions were optimized, and 0.1% formic acid was added in the mobile phase for the better response in positive mode. Compared with isocratic elution, gradient elution could gain shaper peaks and better sensitivity with suitable retention time. Although the chemical structures of analytes were similar, satisfactory separation was achieved using a CORTECS™ C18 column (2.1×50mm, 2.7 *μ*m). Furthermore, there were no endogenous components interfering with the analytes in plasma, as shown in**Figure 3**.

### 3.3. Sample Preparation

The liquid-liquid extraction (LLE) and protein precipitation were evaluated in sample preparation. LLE solvents including chloroform, ethyl acetate, and diethyl ether were investigated, which showed poor recoveries for the analytes. The protein precipitation was tested with methanol, which could get good response and few interference peaks. The satisfactory recovery and extract efficiency of all analytes were obtained by protein precipitation with methanol.

### 3.4. Method Validation

#### 3.4.1. Specificity

The specificity of the method was evaluated by comparing the MRM chromatograms (**Figure 3**) of blank plasma, spiked plasma sample, and rat plasma sample collected 0.5 h after oral administration of the extracts. The retention time for AC, HA, MA, BAC, BHA, BMA, Rb_1_, Rb_2_, Rc, Rd, Re, Rf, Rg_1_, and IS were 10.43, 10.33, 9.26, 5.71, 6.89, 4.04, 11.07, 11.69, 11.37, 12.46, 7.17, 10.16, 7.01, and 8.24 min, respectively. There is no significant interference in the blank rat plasma. The total analysis time of 13 analytes was less than 15 min due to the well-optimized LC system and high selectivity of the MRM mode.

#### 3.4.2. Linearity and Sensitivity

The calibration curves of six* Aconitum* alkaloids and seven ginsenosides were established by 1/*x*^*2*^ weighted linear regression model. The linear calibration range studied was 0.1-50 ng/mL for six* Aconitum* alkaloids and 1-500 ng/mL for seven ginsenosides with coefficients of determination greater than 0.995. The regression equations, linear ranges, coefficients, and LLOQ are listed in**Table 2**.* y* is the IS corrected peak area ratio of the analyte and* x* is the concentration of an analyte in spiked plasma samples.

The LLOQs of the developed method were more sensitive compared to the previously published methods, demonstrating that this method can effectively detect trace level of 13 analytes in plasma.

#### 3.4.3. Precision and Accuracy

The intraday precision, interday precision, and accuracy of the method were determined by analyzing QC samples for each analyte (six replicates per concentration, four concentration levels). All data are listed in**Table 3**. The intra- and interday precisions (RSD) were 0.9-14.3%. The accuracy was within ±14.2%. The results indicate that the developed method is accurate and reproducible.

#### 3.4.4. Extraction Recovery and Matrix Effect

As shown in**Table 4**, the absolute recoveries of the analytes in plasma ranged from 64.2 ± 5.2 to 94.1 ± 2.3% at three concentration levels, while the recovery of IS was 92.2 ± 3.0%. The matrix effects for 13 analytes ranged from 91.2 ± 3.6 to 118.1 ± 6.3% and 85.5 ± 4.1% for IS. In addition, the RSD of the analytes normalized matrix factor was less than 11.5%. The results indicate that the efficiency of protein precipitation is acceptable and the endogenous matrix peaks could not affect the quantification of all the analytes.

#### 3.4.5. Stability

As shown in**Table 5**, the stability of the analytes in rat plasma was investigated under a variety of conditions at three concentrations. All the analytes were confirmed to be stable at room temperature within 3 h (RSD* <*14.1%), in autosampler after preparation for 4 h (RSD* <*11.2%), at −70°C for 7 days (RSD* <*14.1%), and three freeze-thaw cycles (RSD* <*13.1%).

### 3.5. Application

The validated method has been applied to investigate the pharmacokinetic studies of* Aconitum* alkaloids and ginsenosides in rat plasma after oral administration of Shen-Fu prescription at a single dose of 4.75 g/kg.

**Figure 4:**
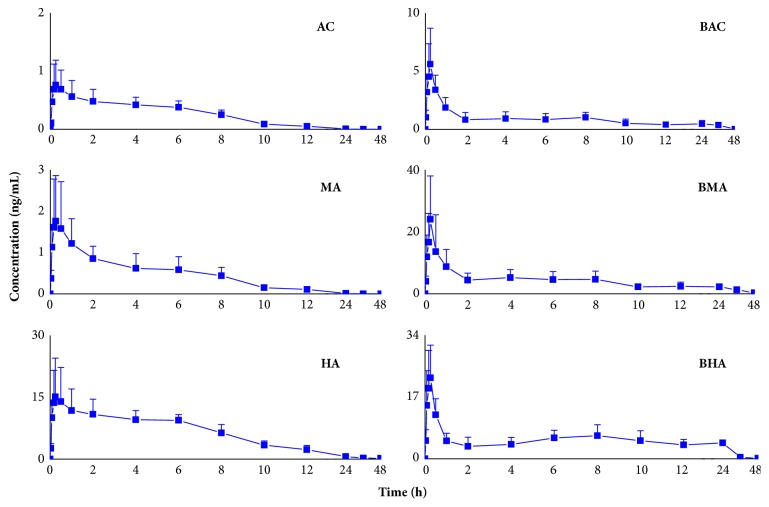
Plasma concentration-time profiles of* Aconitum* alkaloids in rats after oral administration of Shen-Fu prescription at a single dose of 4.75 g/kg to SD rats (mean ± SD, n = 8).

The plasma concentration-time profiles of* Aconitum* alkaloids are presented in**Figure 4**. The major pharmacokinetic parameters are listed in**Table 6**. The time to reach the maximum plasma concentration (Tmax) of AC, HA, MA, BAC, BHA, and BMA was 0.87 ± 0.67 h, 0.87 ± 0.67 h, 0.87 ± 0.67 h, 0.29 ± 0.17 h, 0.18 ± 0.06 h, and 0.25 ± 0.14 h, respectively, suggesting that the absorption of* Aconitum* alkaloids in the plasma was very fast. Meanwhile, elimination half-life (t_1/2_) of AC, HA, MA, BAC, BHA, and BMA was 3.07 ±1.31 h, 4.02 ± 1.02 h, 3.07 ± 2.05 h, 1.10 ± 0.68 h, 0.54 ± 0.37 h, and 1.09± 0.71 h. The results indicate that* Aconitum* alkaloids eliminate rapidly with a short half-life, which is related to the low capacity of protein bounding [8]. In addition, AUC(0-t_n_) of AC, HA, MA, BAC, BHA, and BMA were 4.13 ± 1.28 h·ng/mL, 114.69 ± 21.46 h·ng/mL, 7.33 ± 2.38 h·ng/mL, 23.34 ± 13.01 h·ng/mL, 119.00 ±82.80 h·ng/mL, and 101.72 ± 73.24 h·ng/mL. The AUC(0-t_n_) of monoester-diterpenoid alkaloids were significantly greater than those of diester-diterpenoid alkaloids.

**Figure 5:**
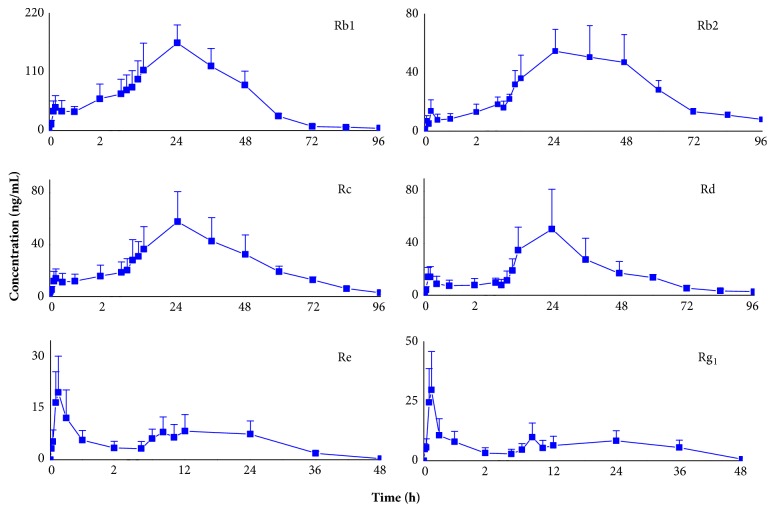
Plasma concentration-time profiles of ginsenosides in rats after oral administration of Shen-Fu prescription at a single dose of 4.75 g/kg to SD rats (mean ± SD, n = 8).

The ppd-type Rb1, Rb2, Rc, and Rd were detectable in rat plasma until 96 h, whereas the ppt-type Re and Rg1 were 48 h. Unlike the above ginsenosides discussed, the ppt-type Rf was below LOQ in plasma at the most time points. The plasma profiles were illustrated in**Figure 5**. In addition, the pharmacokinetic parameters were shown in**Table 6**. It was reported that one peak was observed in the plasma concentration time course of Rb1, Rb2, Rc, and Rd after oral administration of the extract from ginseng [30, 31]. However, double peaks in curves of plasma concentration of ginsenosides were observed in**Figure 5** in this study. The first peak appeared at about 0.25 h, and the second peak appeared at about 24 h. Similarly, double peaks were also observed in the plasma concentration time courses of monoester-diterpenoid alkaloids (BAC, BMA, and BHA), which was different from previous reports [32].

The results of the present research show the pharmacokinetics profiles of six* Aconitum* alkaloids and seven ginsenosides in Shen-Fu prescription simultaneously for the first time. The pharmacokinetics results could provide a useful reference for future research and clinical application.

## 4. Conclusions

A sensitive and reliable LC-MS/MS method with protein precipitation in sample preparation was developed for simultaneous determination of AC, HA, MA, BAC, BHA, BMA, Rb_1_, Rb_2_, Rc, Rd, Re, Rf, and Rg_1_ in rat plasma. With 15 min analysis time, the established method was efficient for the analysis of large numbers of plasma samples. The developed method has been successfully applied to pharmacokinetic study on rats after oral administration of the Shen-Fu prescription at 4.75 mg/kg. The results of the pharmacokinetic study of six* Aconitum* alkaloids and seven ginsenosides could provide valuable data for the clinical use of Shen-Fu prescription.

## Figures and Tables

**Table 1:** Mass spectra properties of the compounds and IS.

Compounds	Precursor Ion (m/z)	Product Ion (m/z)	Frag. (V)	C.E. (V)
AC	646.4	586.3	240	35
MA	632.3	572.2	200	35
HA	616.3	556.2	175	35
BAC	604.3	105.0	140	60
BMA	590.3	105.0	240	60
BHA	574.3	105.0	240	60
Rb_1_	1131.6	364.9	380	70
Rb_2_	1101.6	335.0	370	65
Rc	1101.6	335.0	360	65
Rd	969.6	789.5	350	55
Re	969.6	789.5	340	45
Rf	823.5	365.1	320	55
Rg_1_	823.5	643.4	310	40
Psoralen (IS)	187.0	131.1	115	25

**Table 2:** Regression equations, correlation coefficients, linear ranges, and LLOQ of the 13 analytes.

Compounds	Regression equation	Correlation	Linear range	LLOQ
		coefficients(r)	(ng mL^−1^)	(ng mL^−1^)
AC	y=13.473763x+0.001605	0.9954	0.1-50.0	0.1
MA	y=9.925801x+0.000764	0.9967	0.1-50.0	0.1
HA	y=11.607255x+0.007185	0.9965	0.1-50.0	0.1
BAC	y=8.722507x+0.005974	0.9966	0.1-50.0	0.1
BMA	y=13.233070x+0.019068	0.9976	0.1-50.0	0.1
BHA	y=6.583533x+0.012468	0.9977	0.1-50.0	0.1
Rb_1_	y=0.417970x-0.001883	0.9980	1.0-500.0	1.0
Rb_2_	y=0.494477x-0.004015	0.9970	1.0-500.0	1.0
Rc	y=0.539509x-0.002736	0.9972	1.0-500.0	1.0
Rd	y=0.209590x-0.000697	0.9954	1.0-500.0	1.0
Re	y=0.247590x+0.000176	0.9953	1.0-500.0	1.0
Rf	y=0.232941x-0.002424	0.9975	1.0-500.0	1.0
Rg_1_	y=0.514582x+0.002851	0.9964	1.0-500.0	1.0

**Table 3:** The accuracy and precision of the 13 analytes in rat plasma (*n* = 6).

Compounds	Concentration (ng mL^−1^)	Intraday			Interday		
		Measured concentration (ng mL^−1^)	Accuracy (RE, %)	Precision (RSD, %)	Measured concentration (ng mL^−1^)	Accuracy (RE, %)	Precision (RSD, %)
AC	0.1	0.1 ± 0.01	-6.6	5.9	0.1 ± 0.01	-12.8	7.8
	0.2	0.2 ± 0.03	-0.2	14.3	0.2 ± 0.02	4.9	11.8
	5	4.7 ± 0.19	-6.8	4.1	4.3 ± 0.21	-14.2	4.9
	50	46.7 ± 1.26	-6.7	2.7	46.3 ± 2.27	-7.5	4.9
MA	0.1	0.1 ± 0.01	8.4	9.4	0.1 ± 0.01	-3.6	5.5
	0.2	0.2 ± 0.02	7.3	8.4	0.2 ± 0.02	5.5	10.5
	5	4.4 ± 0.09	-12.1	2.2	4.4 ± 0.11	-12	2.4
	50	44.0 ± 1.54	-12.1	3.5	46.5 ± 4.41	-7.1	9.5
HA	0.1	0.1 ± 0.01	-8.7	7.2	0.1 ± 0.01	3.2	6.0
	0.2	0.2 ± 0.02	6.9	7.8	0.2 ± 0.02	-0.3	12.3
	5	4.5 ± 0.13	-9.2	2.8	4.5 ± 0.13	-9.9	2.9
	50	43.4 ± 0.39	-13.2	0.9	47.6 ± 5.14	-4.8	10.8
BAC	0.1	0.1 ± 0.01	6.2	11.8	0.1 ± 0.01	-1.5	8.7
	0.2	0.2 ± 0.02	-8.5	10.4	0.2 ± 0.02	-3.9	9.1
	5	4.7 ± 0.38	-5.9	8.1	5.0 ± 0.45	-0.5	9.1
	50	46.2 ± 2.03	-7.6	4.4	48.9 ± 4.49	-2.3	9.2
BMA	0.1	0.1 ± 0.01	-12.4	10.0	0.1 ± 0.01	7.6	7.2
	0.2	0.2 ± 0.01	2.4	6.9	0.2 ± 0.01	-2.4	7.4
	5	4.5 ± 0.21	-10.6	4.6	4.8 ± 0.40	-4	8.4
	50	43.9 ± 0.92	-12.2	2.1	47.0 ± 3.81	-6	8.1
BHA	0.1	0.1 ± 0.01	9.8	5.9	0.1 ± 0.01	-4.7	9.7
	0.2	0.2 ± 0.02	-7.6	8.9	0.2 ± 0.02	-0.9	8.7
	5	4.9 ± 0.62	-2.1	12.6	5.2 ± 0.53	3.8	10.3
	50	47.8 ± 3.78	-4.4	7.9	49.9 ± 4.69	-0.2	9.4
Rb_1_	1	1.0 ± 0.09	4.5	8.9	0.9 ± 0.08	-11.5	8.6
	2	2.0 ± 0.18	1.3	8.8	2.1 ± 0.19	3.2	9.0
	50	48.9 ± 3.86	-2.3	7.9	48.8 ± 3.42	-2.4	7.0
	500	458.5 ± 6.88	-8.3	1.5	498.5 ± 44.37	-0.3	8.9
Rb_2_	1	0.9 ± 0.04	-10.4	4.1	1.1 ± 0.08	6.3	7.8
	2	1.9 ± 0.11	-2.8	5.5	1.9 ± 0.20	-3.7	10.2
	50	54.4 ± 2.12	8.7	3.9	48.4 ± 4.79	-3.2	9.9
	500	494.5 ± 46.48	-1.1	9.4	490.0 ± 40.67	-2	8.3
Rc	1	1.0 ± 0.07	3.2	7.0	0.9 ± 0.03	5.3	3.5
	2	1.8 ± 0.11	-11.9	6.3	1.9 ± 0.22	-3.9	11.2
	50	44.9 ± 0.90	-10.2	2.0	48.9 ± 5.08	-2.3	10.4
	500	436.5 ± 10.91	-12.7	2.5	489.5 ± 53.36	-2.1	10.9
Rd	1	1.0 ± 0.03	-1.7	3.3	0.9 ± 0.09	-7.5	9.9
	2	1.8 ± 0.09	-8.4	4.7	1.8 ± 0.17	-7.8	9.3
	50	47.9 ± 1.63	-4.3	3.4	48.8 ± 4.14	-2.5	8.5
	500	447.0 ± 16.09	-10.6	3.6	496.5 ± 48.16	-0.7	9.7
Re	1	0.9 ± 0.07	-7.6	7.1	1.1 ± 0.06	6.3	5.8
	2	1.9 ± 0.14	-3.2	7.1	2.1 ± 0.22	3.2	10.7
	50	48.6 ± 3.11	-2.9	6.4	50.8 ± 4.32	1.6	8.5
	500	461.5 ± 14.77	-7.7	3.2	453.0 ± 20.39	-9.4	4.5
Rf	1	1.1 ± 0.12	9.6	11.0	1.0 ± 0.03	2.3	3.3
	2	1.8 ± 0.11	-9.3	6.2	1.9 ± 0.18	-7.2	9.8
	50	48.5 ± 2.62	-3.1	5.4	50.6 ± 4.09	1.1	8.1
	500	437.0 ± 11.80	-12.6	2.7	514.0 ± 9.11	2.8	11.5
Rg_1_	1	1.0 ± 0.08	2.4	8.2	1.0 ± 0.08	-2.7	7.7
	2	2.1 ± 0.13	5.2	6.2	2.1 ± 0.21	7.3	9.6
	50	49.0 ± 1.96	-2	4.0	52.2 ± 3.75	4.3	7.2
	500	457.0 ± 16.00	-8.6	3.5	458.0 ± 19.24	-8.4	4.2

**Table 4:** The mean recovery and matrix effect of the 13 analytes in rat plasma (*n* = 6).

Compounds	Concentration	Absolute recovery	RSD	Matrix effect	RSD
	(ng mL^−1^)	(%)	(%)	(%)	(%)
AC	0.2	76.2 ± 2.8	3.7	96.8 ± 7.3	7.5
	5	71.3 ± 1.6	2.2	101.2 ± 2.9	2.9
	50	72.5 ± 1.4	1.9	96.9 ± 1.7	1.7
MA	0.2	76.6 ± 6.2	8.1	98.3 ± 11.3	11.5
	5	70.7 ± 1.6	2.3	100.2 ± 2.6	2.6
	50	71.3 ± 2.1	2.9	99.8 ± 1.8	1.8
HA	0.2	70.1 ± 5.3	7.6	98.3 ± 8.3	8.5
	5	66.1 ± 1.7	2.6	95.3 ± 6.1	6.4
	50	68.2 ± 1.6	2.3	99.0 ± 1.6	1.6
BAC	0.2	75.4 ± 4.6	6.1	95.3 ± 5.9	6.2
	5	71.2 ± 2.1	2.9	98.3 ± 6.1	6.2
	50	69.8 ± 6.1	8.7	95.5 ± 3.4	3.6
BMA	0.2	79.3 ± 4.7	5.9	97.1 ± 6.0	6.2
	5	76.4 ± 5.1	6.7	99.0 ± 4.6	4.6
	50	73.3 ± 2.9	4.0	99.3 ± 1.9	1.9
BHA	0.2	82.1 ± 3.7	4.5	101.2 ± 4.2	4.1
	5	76.9 ± 4.2	5.5	99.7 ± 5.0	4.9
	50	78.3 ± 5.8	7.4	91.2 ± 3.6	3.9
Rb_1_	2	89.2 ± 6.1	6.8	101.3 ± 5.5	5.5
	50	94.1 ± 2.3	2.4	97.8 ± 4.2	4.3
	500	93.2 ± 4.1	4.4	95.0 ± 5.7	6.0
Rb_2_	2	88.9 ± 6.2	7.0	102.0 ± 5.2	5.1
	50	90.1 ± 3.0	3.3	95.1 ± 4.8	5.1
	500	92.1 ± 1.7	1.8	97.4 ± 5.5	5.7
Rc	2	85.1 ± 6.2	7.3	97.4 ± 5.7	5.9
	50	88.3 ± 4.8	5.4	100.3 ± 3.6	3.6
	500	90.6 ± 4.5	5.0	103.7 ± 5.5	5.3
Rd	2	91.2 ± 7.1	7.8	106.3 ± 7.8	7.3
	50	86.9 ± 3.1	3.6	112.8 ± 5.6	5.0
	500	89.1 ± 5.2	5.8	114.2 ± 7.2	6.3
Re	2	68.7 ± 2.3	3.3	113.1 ± 8.9	7.9
	50	64.2 ± 5.2	8.1	116.1 ± 5.2	4.5
	500	66.1 ± 1.6	2.4	114.4 ± 5.1	4.5
Rf	2	74.5 ± 5.9	7.9	112.5 ± 6.2	5.5
	50	71.6 ± 4.8	6.7	118.1 ± 6.3	5.3
	500	73.2 ± 2.6	3.6	115.6 ± 2.8	2.4
Rg_1_	2	71.3 ± 4.2	5.9	117.1 ± 4.4	3.8
	50	66.4 ± 5.8	8.7	113.6 ± 2.9	2.6
	500	68.1 ± 2.8	4.1	114.7 ± 5.3	4.6
IS	200	92.2 ± 3.0	3.3	85.5 ± 4.1	4.8

**Table 5:** Stability of the 13 analytes in rat plasma (n=3).

Compounds and Concentration (ng mL^−1^)		3 h at room temperature		4 h in autosampler		3 freeze-thaw cycles		7 days of storage at -70°C	
		Measured Concentration (ng mL^−1^)	RSD (%)	Measured Concentration (ng mL^−1^)	RSD (%)	Measured Concentration (ng mL^−1^)	RSD (%)	Measured Concentration (ng mL^−1^)	RSD (%)
AC	0.2	0.2 ± 0.03	14.1	0.2 ± 0.01	5.8	0.2 ± 0.01	4.1	0.2 ± 0.02	10.3
	5	5.2 ± 0.2	3.0	4.7 ± 0.1	1.2	4.5 ± 0.2	4.6	4.4 ± 0.1	3.1
	50	55.2 ± 1.9	3.5	44.2 ± 1.4	3.1	45.6 ± 1.0	2.3	55.9 ± 1.1	2.0
MA	0.2	0.2 ± 0.01	3.1	0.2 ± 0.01	2.9	0.2 ± 0.02	9.8	0.2 ± 0.01	2.8
	5	4.9 ± 0.1	1.3	4.4 ± 0.1	2.7	4.4 ± 0.02	0.4	4.6 ± 0.3	6.6
	50	54.3 ± 1.4	2.5	44.7 ± 1.0	2.3	46.9 ± 1.5	3.2	55.8 ± 1.9	3.3
HA	0.2	0.2 ± 0.003	1.6	0.2 ± 0.02	8.0	0.2 ± 0.01	3.7	0.2 ± 0.02	10.2
	5	5.2 ± 0.2	3.3	4.4 ± 0.1	2.2	4.6 ± 0.1	2.8	4.6 ± 0.1	3.0
	50	54.3 ± 0.7	1.2	45.1 ± 1.8	4.0	49.5 ± 1.6	3.1	55.5 ± 1.3	2.3
BAC	0.2	0.2 ± 0.02	8.6	0.2 ± 0.02	9.9	0.2 ± 0.01	7.2	0.2 ± 0.01	5.9
	5	4.9 ± 0.4	8.7	4.4 ± 0.1	2.9	5.5 ± 0.3	5.7	4.8 ± 0.4	8.9
	50	48.8 ± 6.8	13.9	47.7 ± 2.6	5.5	51.8 ± 6.8	13.1	50.6 ± 7.1	14.1
BMA	0.2	0.2 ± 0.01	6.2	0.2 ± 0.01	3.4	0.2 ± 0.01	7.2	0.2 ± 0.02	9.2
	5	5.0 ± 0.1	2.6	4.6 ± 0.5	11.2	4.6 ± 0.1	1.7	5.0 ± 0.3	6.8
	50	51.4 ± 2.9	5.6	44.8 ± 2.1	4.8	50.6 ± 2.7	5.4	52.2 ± 1.3	2.5
BHA	0.2	0.2 ± 0.02	8.6	0.2 ± 0.02	9.9	0.2 ± 0.01	7.2	0.2 ± 0.01	5.9
	5	4.9 ± 0.4	8.7	4.4 ± 0.1	2.9	5.5 ± 0.3	5.7	4.8 ± 0.4	8.9
	50	48.8 ± 6.8	13.9	47.7 ± 2.6	5.5	51.8 ± 6.8	13.1	50.6 ± 7.1	14.1
Rb_1_	2	2.1 ± 0.2	7.8	2.0 ± 0.1	5.7	2.0 ± 0.1	6.9	2.1 ± 0.2	7.4
	50	53.1 ± 1.8	3.4	51.1 ± 2.0	4.0	47.9 ± 5.3	10.9	50.6 ± 5.5	10.8
	500	447.2 ± 5.5	1.2	449.8 ± 15.4	3.4	477.6 ± 56.3	11.8	434.3 ± 6.6	1.5
Rb_2_	2	1.9 ± 0.2	9.0	1.8 ± 0.02	1.2	1.8 ± 0.1	4.4	1.9 ± 0.3	13.2
	50	50.3 ± 6.6	13.1	44.6 ± 0.6	1.4	55.2 ± 0.6	1.1	45.6 ± 4.4	9.7
	500	481.4 ± 20.4	4.2	456.9 ± 10.4	2.3	442.1 ± 20.5	4.6	436.2 ± 16.1	3.7
Rc	2	1.8 ± 0.1	4.3	1.8 ± 0.1	4.7	1.8 ± 0.1	3.7	1.8 ± 0.1	6.2
	50	47.5 ± 4.6	9.8	44.8 ± 1.2	2.7	52.2 ± 2.2	4.1	48.8 ± 6.6	13.6
	500	542.9 ± 20.0	3.7	445.3 ± 13.5	3.0	437.8 ± 9.8	2.2	494.7 ± 17.5	3.5
Rd	2	1.8 ± 0.04	2.3	2.0 ± 0.2	7.8	2.0 ± 0.2	8.3	1.9 ± 0.1	7.9
	50	51.0 ± 4.6	9.1	45.7 ± 1.7	3.6	48.6 ± 2.3	4.7	43.7 ± 0.6	1.3
	500	564.6 ± 7.5	1.3	443.4 ± 7.2	1.6	443.4 ± 11.8	2.7	501.0 ± 21.3	4.2
Re	2	2.1 ± 0.1	7.2	1.8 ± 0.1	4.8	1.9 ± 0.2	11.9	1.9 ± 0.2	12.0
	50	56.4 ± 0.9	1.6	50.5 ± 1.4	2.7	52.8 ± 3.8	7.1	52.0 ± 6.4	12.2
	500	437.3 ± 11.7	2.7	468.4 ± 7.6	1.6	454.6 ± 23.6	5.2	443.1 ± 19.5	4.4
Rf	2	2.1 ± 0.1	4.5	1.9 ± 0.2	8.7	1.8 ± 0.1	5.0	1.9 ± 0.2	12.2
	50	47.8 ± 4.0	8.4	47.8 ± 1.9	4.0	53.2 ± 2.7	5.0	49.5 ± 3.0	6.1
	500	553.4 ± 10.6	1.9	440.2 ± 12.2	2.8	439.0 ± 9.8	2.2	506.5 ± 22.9	4.5
Rg_1_	2	2.0 ± 0.1	5.5	1.8 ± 0.05	2.7	1.9 ± 0.1	7.5	2.1 ± 0.1	5.1
	50	55.6 ± 0.5	0.9	51.5 ± 1.9	3.8	55.9 ± 0.7	1.3	52.7 ± 4.2	8.0
	500	456.7 ± 16.9	3.7	461.0 ± 8.1	1.8	439.8 ± 20.7	4.7	437.8 ± 2.5	0.6

**Table 6:** Pharmacokinetic parameters of the six *Aconitum* alkaloids and seven ginsenosides after oral administration of Shen-Fu prescription (n=8).

Compounds	T_max_ (h)	C_max_ (ng mL^−1^)	t_1/2_ (h)	AUC_(0-tn)_ (h·ng mL^−1^)	AUC_(0-*∞*)_ (h·ng mL^−1^)	MRT_(0-t)_ (h)	MRT_(0-*∞*)_ (h)
AC	0.87 ± 0.67	0.91 ± 0.40	3.07 ± 1.31	4.13 ± 1.28	4.26 ± 1.36	4.65 ± 1.31	5.22 ± 1.57
MA	0.87 ± 0.67	2.00 ± 1.15	3.07 ± 2.05	7.33 ± 2.38	7.97 ± 2.01	4.34 ± 1.10	4.79 ± 2.58
HA	0.87 ± 0.67	17.96 ± 6.89	4.02 ± 1.02	114.69 ± 21.46	123.54 ± 28.09	7.47 ± 2.74	8.16 ± 4.95
BAC	0.29 ± 0.17	6.56 ± 3.32	1.10 ± 0.68	23.34 ± 13.01	24.93 ± 14.82	12.29 ± 6.23	14.14 ± 6.27
BMA	0.25 ± 0.14	27.40 ± 16.27	1.09 ± 0.71	101.72 ± 73.24	114.96 ± 82.38	9.39 ± 4.70	10.34 ± 5.10
BHA	0.18 ± 0.06	28.96 ± 15.83	0.54 ± 0.37	119.00 ±82.80	123.92 ± 80.97	8.98 ± 4.21	10.11 ± 3.90
Rb_1_	22.91 ± 3.62	164.85 ± 33.22	14.29 ± 1.60	6536.09 ± 1602.23	6601.72 ± 1606.56	30.37 ± 1.37	31.58 ± 1.61
Rb_2_	28.00 ± 6.20	59.70 ± 15.60	19.65 ± 3.24	2965.44 ± 752.25	3167.67 ± 700.86	39.77 ± 2.51	50.06 ± 8.34
Rc	22.54 ± 4.82	58.98 ± 23.16	16.97 ± 2.02	2558.56 ± 824.52	2614.91 ± 825.03	35.98 ± 2.09	37.83 ± 2.36
Rd	22.29 ± 4.54	51.23 ± 30.78	17.68 ± 8.31	1794.84 ± 839.20	1868.61 ± 820.37	35.27± 5.49	40.37 ± 10.10
Re	0.26 ± 0.12	27.72 ± 10.22	14.62 ± 9.40	242.21 ± 97.15	278.46 ± 87.95	16.49 ± 2.63	19.96 ± 3.35
Rg_1_	0.24 ± 0.15	37.61 ± 18.98	15.82 ± 10.83	242.85 ± 172.14	261.81 ± 168.63	16.26 ± 6.73	19.58 ± 9.00

## Data Availability

The data used to support the findings of this study are available from the corresponding author upon request.

## References

[1] Luo J., Min S., Wei K., Cao J. (2008). Ion channel mechanism and ingredient bases of Shenfu Decoction's cardiac electrophysiological effects. *Journal of Ethnopharmacology*.

[2] Wang Y.-L., Wang C.-Y., Zhang B.-J., Zhang Z.-Z. (2009). Shenfu injection suppresses apoptosis by regulation of Bcl-2 and caspase-3 during hypoxia/reoxygenation in neonatal rat cardiomyocytes in vitro. *Molecular Biology Reports*.

[3] Xiao Y., Ma Z.-C., Wang Y.-G. (2013). Cardioprotection of Shenfu preparata on cardiac myocytes through cytochrome P450 2J3.. *Journal of Integrative Medicine*.

[4] Singhuber J., Zhu M., Prinz S., Kopp B. (2009). Aconitum in traditional Chinese medicine: a valuable drug or an unpredictable risk?. *Journal of Ethnopharmacology*.

[5] Gao T., Bi H., Ma S., Lu J. (2010). The antitumor and immunostimulating activities of water soluble polysaccharides from Radix Aconiti, Radix Aconiti Lateralis and Radix Aconiti Kusnezoffii. *Natural Product Communications (NPC)*.

[6] Liu J., Li Q., Yin Y., Liu R., Xu H., Bi K. (2014). Ultra-fast LC-ESI-MS/MS method for the simultaneous determination of six highly toxic Aconitum alkaloids from Aconiti kusnezoffii radix in rat plasma and its application to a pharmacokinetic study. *Journal of Separation Science*.

[7] Yu B., Cao Y., Xiong Y.-K. (2015). Pharmacokinetics of aconitine-type alkaloids after oral administration of Fuzi (Aconiti Lateralis Radix Praeparata) in rats with chronic heart failure by microdialysis and ultra-high performance liquid chromatography-tandem mass spectrometry. *Journal of Ethnopharmacology*.

[8] Tang L., Gong Y., Lv C., Ye L., Liu L., Liu Z. (2012). Pharmacokinetics of aconitine as the targeted marker of fuzi (*Aconitum carmichaeli*) following single and multiple oral administrations of fuzi extracts in rat by UPLC/MS/MS. *Journal of Ethnopharmacology*.

[9] Chan T. Y. K., Tomlinson B., Tse L. K. K., Chan J. C. N., Chan W. W. M., Critchley J. A. J. H. (1994). Aconitine poisoning due to Chinese herbal medicines: A review. *Veterinary and Human Toxicology*.

[10] Tai Y.-T., Lau C.-P., Young K., But P. P.-H. (1992). Cardiotoxicity after accidental herb-induced aconite poisoning. *The Lancet*.

[11] Chen J.-H., Lee C.-Y., Liau B.-C., Lee M.-R., Jong T.-T., Chiang S.-T. (2008). Determination of aconitine-type alkaloids as markers in fuzi (*Aconitum carmichaeli*) by LC/(+)ESI/MS3. *Journal of Pharmaceutical and Biomedical Analysis*.

[12] Guo N., Liu M., Yang D.-W. (2013). Quantitative LC-MS/MS analysis of seven ginsenosides and three aconitum alkaloids in Shen-Fu decoction. *Chemistry Central Journal*.

[13] Kang S., Min H. (2012). Ginseng, the 'immunity boost': The effects of panax ginseng on immune system. *Journal of Ginseng Research*.

[14] Hu C., Kitts D. D. (2001). Free radical scavenging capacity as related to antioxidant activity and ginsenoside composition of Asian and North American Ginseng extracts. *Journal of the American Oil Chemists’ Society*.

[15] Zhou H., Hou S. Z., Luo P. (2011). Ginseng protects rodent hearts from acute myocardial ischemia-reperfusion injury through GR/ER-activated RISK pathway in an endothelial NOS-dependent mechanism. *Journal of Ethnopharmacology*.

[16] He J., Zhao J., Ma Z. (2015). Serum Pharmacochemistry Analysis Using UPLC-Q-TOF/MS after Oral Administration to Rats of Shenfu Decoction. *Evidence-Based Complementary and Alternative Medicine*.

[17] Wang X. L., Li L. J., Li Y. M., Li C. Y., Zhang D. F. (2015). Toxicity-reducing effect of compatibility of *Aconiti Lateralis*radix praeparata with different proportion of* Panax Ginseng* in neonatal rat cardiomyocytes. *Chinese Journal of Experimental Traditional Medical Formulae*.

[18] Ye R., Yang Q., Kong X. (2011). Ginsenoside Rd attenuates early oxidative damage and sequential inflammatory response after transient focal ischemia in rats. *Neurochemistry International*.

[19] Ye R., Zhang X., Kong X. (2011). Ginsenoside Rd attenuates mitochondrial dysfunction and sequential apoptosis after transient focal ischemia. *Neuroscience*.

[20] Wang X., Wang H., Zhang A. (2012). Metabolomics study on the toxicity of aconite root and its processed products using ultraperformance liquid-chromatography/electrospray-ionization synapt high-definition mass spectrometry coupled with pattern recognition approach and ingenuity pathways analysis. *Journal of Proteome Research*.

[21] Bao Y., Yang F., Yang X. (2011). CE-electrochemiluminescence with ionic liquid for the facile separation and determination of diester-diterpenoid aconitum alkaloids in traditional Chinese herbal medicine. *Electrophoresis*.

[22] Wang J., van der Heijden R., Spijksma G. (2009). Alkaloid profiling of the Chinese herbal medicine Fuzi by combination of matrix-assisted laser desorption ionization mass spectrometry with liquid chromatography-mass spectrometry. *Journal of Chromatography A*.

[23] Qi X., Ignatova S., Luo G. (2010). Preparative isolation and purification of ginsenosides Rf, Re, Rd and Rb1 from the roots of Panax ginseng with a salt/containing solvent system and flow step-gradient by high performance counter-current chromatography coupled with an evaporative light scattering detector. *Journal of Chromatography A*.

[24] Li X., Wang G., Sun J. (2007). Pharmacokinetic and absolute bioavailability study of total Panax notoginsenoside, a typical multiple constituent traditional Chinese medicine (TCM) in rats. *Biological & Pharmaceutical Bulletin*.

[25] Song Y. L., Zhang N., Shi S. P. (2015). Large-scale qualitative and quantitative characterization of components in Shenfu injection by integrating hydrophilic interaction chromatography, reversed phase liquid chromatography, and tandem mass spectrometry. *Journal of Chromatography A*.

[26] Li Z., Zhang R., Wang X., Hu X., Chen Y., Liu Q. (2015). Simultaneous determination of seven ginsenosides in rat plasma by high-performance liquid chromatography coupled to time-of-flight mass spectrometry: application to pharmacokinetics of Shenfu injection. *Biomedical Chromatography*.

[27] Zhang F., Tang M.-H., Chen L.-J. (2008). Simultaneous quantitation of aconitine, mesaconitine, hypaconitine, benzoylaconine, benzoylmesaconine and benzoylhypaconine in human plasma by liquid chromatography-tandem mass spectrometry and pharmacokinetics evaluation of ‘SHEN-FU’ injectable powder. *Journal of Chromatography B*.

[28] Ma Z. C., Zhou S. S., Liang Q. D. (2011). UPLC-TOF/MS based chemical profiling approach to evaluate toxicity-attenuated chemical composition in combination of ginseng and Radix Aconiti Praeparata. *Acta Pharmaceutica Sinica*.

[29] Zhang Y., Tian D., Huang Y. (2016). Pharmacokinetic evaluation of Shenfu Injection in beagle dogs after intravenous drip administration. *Acta Pharmaceutica Sinica B (APSB)*.

[30] Kang A., Qian J., Shan J.-J., Di L.-Q. (2015). In vivo pharmacokinetic study on total saponins from roots of Panax ginseng in rats. *Chinese Traditional and Herbal Drugs*.

[31] Liu H., Yang J., Du F. (2009). Absorption and disposition of ginsenosides after oral administration of Panax notoginseng extract to rats. *Drug Metabolism and Disposition*.

[32] Peng W.-W., Li W., Li J.-S. (2013). The effects of Rhizoma Zingiberis on pharmacokinetics of six *Aconitum alkaloids* in herb couple of Radix Aconiti Lateralis-Rhizoma Zingiberis. *Journal of Ethnopharmacology*.

